# Early Segmental White Matter Fascicle Microstructural Damage Predicts the Corresponding Cognitive Domain Impairment in Cerebral Small Vessel Disease Patients by Automated Fiber Quantification

**DOI:** 10.3389/fnagi.2020.598242

**Published:** 2021-01-11

**Authors:** Lili Huang, Xin Chen, Wenshan Sun, Haifeng Chen, Qing Ye, Dan Yang, Mengchun Li, Caimei Luo, Junyi Ma, Pengfei Shao, Hengheng Xu, Bing Zhang, Xiaolei Zhu, Yun Xu

**Affiliations:** ^1^Department of Neurology, Drum Tower Hospital, Medical School and the State Key Laboratory of Pharmaceutical Biotechnology, Institute of Brain Science, Nanjing University, Nanjing, China; ^2^Jiangsu Key Laboratory for Molecular Medicine, Medical School of Nanjing University, Nanjing, China; ^3^Jiangsu Province Stroke Center for Diagnosis and Therapy, Nanjing, China; ^4^Nanjing Neurological Medical Center, Nanjing, China; ^5^Department of Radiology, Affiliated Drum Tower Hospital of Nanjing University Medical School, Nanjing, China

**Keywords:** automated fiber quantification, cerebral small vessel disease, cognitive decline, damage mode, white matter microstructure damage

## Abstract

**Objective:** To characterize earlier damage pattern of white matter (WM) microstructure in cerebral small vessel disease (CSVD) and its relationship with cognitive domain dysfunction.

**Methods:** A total of 144 CSVD patients and 100 healthy controls who underwent neuropsychological measurements and diffusion tensor imaging (DTI) examination were recruited. Cognitive function, emotion, and gait were assessed in each participant. The automated fiber quantification (AFQ) technique was used to extract different fiber properties between groups, and partial correlation and general linear regression analyses were performed to assess the relationship between position-specific WM microstructure and cognitive function.

**Results:** Specific segments in the association fibers, commissural WM regions of interest (ROIs), and projection fibers were damaged in the CSVD group [*P* < 0.05, family-wise error (FWE) correction], and these damaged segments showed interhemispheric symmetry. In addition, the damage to specific tract profiles [including the posteromedial component of the right cingulum cingulate (CC), the occipital lobe portion of the callosum forceps major, the posterior portion of the left superior longitudinal fasciculus (SLF), and the bilateral anterior thalamic radiation (ATR)] was related to the dysfunction in specific cognitive domains. Among these tracts, we found the ATR to be the key set of tracts whose profiles were most associated with cognitive dysfunction. The left ATR was a specific fiber bundle associated with episode memory and language function, whereas the fractional anisotropy (FA) values of the intermediate component of the right ATR were negatively correlated with executive function and gait evaluation. It should be noted that the abovementioned relationships could not survive the Bonferroni correction (*p* < 0.05/27), so we chose more liberal uncorrected statistical thresholds.

**Conclusions:** Damage to the WM fiber bundles showed extensive interhemispheric symmetry and was limited to particular segments in CSVD patients. Disruption of strategically located fibers was associated with different cognitive deficits, especially the bilateral ATR.

## Introduction

Most cerebral small vessel diseases (CSVDs) are associated with age and hypertension. One of the clinical manifestations of the CSVD is cognitive impairment, which may ultimately progress to dementia (Dichgans et al., 2016). The prevalence of dementia in association with CSVD is as high as 36–67% (Roh and Lee, 2014). Deficits in general cognitive function and other cognitive domains, especially information processing speed and executive function, are common in CSVD patients (Prins et al., 2005; Lawrence et al., 2014). The MRI findings in CSVD include white matter hyperintensities (WMH), lacunar infarction (LI), microbleeds, perivascular spaces, and brain atrophy (Wardlaw et al., 2013). A quantitatively dependent relationship was found between the number of lacunas/WMH and the degree of cognitive impairment (CI; Prins and Scheltens, 2015), but variations in clinical symptoms did not completely correspond to conventional MRI markers (Ter Telgte et al., 2018).

Predicting the progression of cognitive decline and its mechanism in the context of CSVD needs to be studied. Increasing evidence has shown a significant association between CSVD's radiological manifestations and dementia pathology. Magnetic resonance-based diffusion tensor imaging (DTI) provides a measure of the diffusion of water in white matter (WM) tracts, allowing researchers to examine the integrity of axonal pathways in patients with CSVD (Beaulieu, 2002). Damage to the microstructural integrity of the WM tract visible in DTI always precedes macroscopic lesions and the destruction of fibers connecting key brain regions (De Groot et al., 2013). There are two parameters to evaluate microstructural integrity of the WM tract, including mean diffusivity (MD), a measure of water diffusion averaged in all spatial directions, and fractional anisotropy (FA), which provides information about the directionality of water diffusion. Loss of microstructural integrity is typically accompanied by a decrease in FA and/or an increase in MD. Increasing evidence has indicated that DTI parameters are early markers of cognitive decline than volume measures (Nir et al., 2013). Voxel-based analysis (VBA) and tract-based spatial statistics (TBSS) are two measures of DTI analysis that have been used to investigate abnormalities in WM diffusion properties in CSVD studies (Della Nave et al., 2007; Papma et al., 2014; Tuladhar et al., 2015). These studies showed that WMH was associated with an increased MD value, when compared with healthy controls, in multiple brain regions, including the corpus callosum, internal and external capsule, cingulum bundle, and other fibers (Papma et al., 2014; Tuladhar et al., 2015). However, these two analysis techniques have drawbacks. It is difficult for VBA to co-register all subjects accurately in a common space due to the huge variations in the shape of the WM fiber bundles among subjects (Wassermann et al., 2011; Pasi et al., 2016). Although TBSS is more accurate and specific than VBA, it fails to precisely define the actual tract location of fibers in an individual (Bach et al., 2014; Pasi et al., 2016). In addition, spurious effects in multiple skeleton points make the results of TBSS less accurate (Pasi et al., 2016).

The new, automated fiber quantification (AFQ) method could capitalize on the precision of tractography for automatically identifying and localizing fiber tracts in individual brains (Yeatman et al., 2012; Chen et al., 2020). In this study, AFQ was applied to evaluate tract profiles of WM integrity in CSVD patients compared with normal controls. FA and MD were extracted at 100 locations along 20 fiber tracts to find damaged fiber tract segments that cause disruption of neural circuits and the locations that are responsible for certain clinical manifestations. These tract profiles visible on DTI may be early imaging markers for CSVD patients with cognitive decline and may provide possible anatomical mechanisms. In addition, the identification of these location-specific properties will offer us a new target for treatment.

## Materials and Methods

### Study Population

One hundred and forty-four patients with CSVD on MRI were consecutively enrolled between January 2017 and January 2019 from the Department of Neurology of the Affiliated Drum Tower Hospital of Nanjing University Medical School, China. Based on the established research criteria, CSVD is defined as lesions of moderate-to-severe WMH (Prins and Scheltens, 2015; Fazekas scores of 2 or higher) and/or anatomically appropriate lacunar infarcts on neuroimaging, with or without perivascular spaces, microbleeds, and brain atrophy (Wardlaw et al., 2013; Lawrence et al., 2014). The symptoms of CSVD include acute symptoms (e.g., lacunar syndromes and transient ischemic attack) or subacute manifestations (e.g., cognitive impairment, motor disturbances, and emotional disorders). The inclusion criteria were being patients aged between 45 and 85 years and a diagnosis of CSVD based on neuroimaging (WMH and/or lacunes). The exclusion criteria included the presence of any of the following: (1) cerebral infarctions larger than 15 mm in diameter; (2) non-small vascular disease WMH mimics (e.g., multiple sclerosis); (3) intracranial or extracranial large artery stenosis >50%; (4) intracranial hemorrhage; (5) other diseases interfering with neuropsychological tests such as Alzheimer's disease, Parkinson's disease or other psychiatric disorders; (6) MRI contraindications or claustrophobia; and (7) left-handedness. We also recruited 100 healthy individuals from a free clinic or the community during the same time for the healthy control (HC) group. The inclusion criteria comprised: (1)without clinical complaints of cognitive impairment; (2)the scores of clinical scales were within the normal range; (3) without CSVD lesions in MRI. This study was approved by the Ethics Committee of Nanjing Drum Tower Hospital, and written informed consent was obtained from all participants.

### Magnetic Resonance Imaging Scanning

All MRI scans were conducted using a Philips 3.0-T scanner (Achieva 3.0 T TX, Philips Medical Systems, The Netherlands) equipped with an eight-channel head coil to diminish scanner noise and restrict head motion. A three-dimensional, high-resolution, T_1_-weighted turbo gradient echo sequence was performed with the following parameters: repetition time (TR) = 9.8 ms, echo time (TE) = 4.6 ms, flip angle (FA) = 8°, slices = 192, field of view (FOV) = 250 × 250 mm^2^, acquisition matrix = 256 × 256, and thickness = 1.0 mm. Fluid-attenuated inversion recovery (FLAIR) images were obtained with TR/TE/inversion time (TI) of 4,500/333/1,600 ms, number of slices was 200, voxel size was 0.95 × 0.95 × 0.95 mm^3^, and acquisition matrix was 270 × 260. Diffusion tensor images were acquired by spin-echo echo planar imaging (TR = 9154 ms, TE = 55 ms, FOV = 224 ×224 mm^2^, matrix = 112 ×112, slice thickness = 2.5 mm, voxel size = 2 ×2 ×2.5 mm^3^, with 32 directional diffusion encoding (b = 1,000 s/mm^2^), and an acquisition without diffusion encoding (b = 0 s/mm^2^).

### Conventional Magnetic Resonance Imaging Analysis

The number of LIs was counted on T_1_-weighted images and FLAIR sequences by two expert neurologists. The WMH volume, gray matter volume, and intracranial volume (ICV) for each subject was obtained from AccuBrain, a multi-atlas-based anatomical segmentation tool (Wang et al., 2019). The volumes of WMHs and gray matter were divided by the ICV for each participant as a process of normalization (brain volume/ICV ×100 %).

### Magnetic Resonance Image Preprocessing

For the three-dimensional (3D) T_1_ anatomical scans, brain extraction was first performed to delete non-brain structures by a brain extraction tool (BET, a tool in the FSL software). We then used the FMRIB Software Library (FSL version 5.0.9, 1; https://www.fmrib.ox.ac.uk/fsl/) to average and rotate T_1_ images to align with the AC–PC plane. Raw DTI images were also initially preprocessed by BET to remove non-brain tissue, and then eddy current distortions and head motion were corrected by an affine image registration and eddy current program (tools in the FSL software). Whole-brain diffusion metrics were calculated at each voxel by the DTIFIT program (command in the FSL software), producing FA and MD values.

### Automated Fiber Quantification

We used the AFQ software to reconstruct 20 WM fiber tracts in the brain of each subject (Yeatman et al., 2012). The automated calculation was performed by MATLAB R2012b (MathWorks, Inc., Natick, Massachusetts, USA). The AFQ pipeline mainly includes six steps, which were described in Yeatman's paper (Yeatman et al., 2012). The first step is whole-brain deterministic tractography, which is performed on all voxels whose FA > 0.3 in a WM mask. Termination of the tracking occurs when the FA value is < 0.2 or the minimum angle between two consecutive tractography steps is more than 30 degrees. Second, the fiber tract is segmented using waypoint regions of interest (ROIs). Third, the identified fiber tract is refined based on the fiber tract probability maps (Hua et al., 2008). The fourth step is fiber tract cleaning, which filters out fibers more than four standard deviations (SDs) above the mean fiber length or those deviating >5 SDs from the core of the fiber bundle. Fifth, the fiber bundles are clipped at each waypoint ROI to determine the core of the tract. Finally, in the sixth step, 100 equidistant nodes along each tract core are segmented, and diffusivity metrics are extracted using spline interpolation. The 20 identified WM tracts are listed in Table 1. However, we failed to track some fibers, especially the bilateral cingulum hippocampus (CH) and bilateral arcuate fasciculus (AF), because the threshold setting in fiber tracking makes it difficult to identify the tracts adjacent to the gray matter (Johnson et al., 2014; Chen et al., 2020). Therefore, we excluded those four fiber tracts from the analyses.

**Table 1:** Successful identification rate for each of 20 fiber tracts in HC and CSVD groups.

**Index**	**Tract**	**Total subjects or raw samples (N0)**			**No. of subjects showing successful tract identification (N1)**			**Ratio (N1/N0)**		
		**HC**	**CSVD**	**Total**	**HC**	**CSVD**	**Total**	**HC**	**CSVD**	**Total**
1	ATR_L	100	144	244	100	144	244	100.00%	100.00%	100.00%
2	ATR_R	100	144	244	100	144	244	100.00%	100.00%	100.00%
3	CST_L	100	144	244	100	144	244	100.00%	100.00%	100.00%
4	CST_R	100	144	244	100	144	244	100.00%	100.00%	100.00%
5	CC_L	100	144	244	100	144	244	100.00%	100.00%	100.00%
6	CC_R	100	144	244	100	144	244	100.00%	100.00%	100.00%
7	CH_L	100	144	244	78	105	183	78.00%	72.92%	75.00%
8	CH_R	100	144	244	58	66	124	58.00%	45.83%	86.11%
9	Forceps major	100	144	244	100	144	244	100.00%	100.00%	100.00%
10	Forceps minor	100	144	244	100	144	244	100.00%	100.00%	100.00%
11	IFOF_L	100	144	244	100	144	244	100.00%	100.00%	100.00%
12	IFOF_R	100	144	244	100	144	244	100.00%	100.00%	100.00%
13	ILF_L	100	144	244	100	144	244	100.00%	100.00%	100.00%
14	ILF_R	100	144	244	100	144	244	100.00%	100.00%	100.00%
15	SLF_L	100	144	244	100	144	244	100.00%	100.00%	100.00%
16	SLF_R	100	144	244	100	144	244	100.00%	100.00%	100.00%
17	UF_L	100	144	244	100	144	244	100.00%	100.00%	100.00%
18	UF_R	100	144	244	100	144	244	100.00%	100.00%	100.00%
19	AF_L	100	144	244	94	138	232	94.00%	95.83%	95.08%
20	AF_R	100	144	244	88	127	215	88.00%	88.19%	88.11%

*HC, health control; CSVD, cerebral small vessel disease; ATR_L, left anterior thalamic radiation; ATR_R, right anterior thalamic radiation; CST_L, left corticospinal tract; CST_R, right corticospinal tract; CC_L, left cingulum cingulate; CC_R, right cingulum cingulate; CH_L, left cingulum hippocampus; CH_R, right cingulum hippocampus; IFOF_L, left inferior fronto-occipital fasciculus; IFOF_R, right inferior fronto-occipital fasciculus; ILF_L, left inferior longitudinal fasciculus; ILF_R, right inferior longitudinal fasciculus; SLF_L, left superior longitudinal fasciculus; SLF_R, right superior longitudinal fasciculus; UF_L, left uncinate fasciculus; UF_R, right uncinate fasciculus; AF_L, left arcuate fasciculus; AF_R, right arcuate fasciculus*.

### Neuropsychological Measurements

The neuropsychological evaluation protocol for each subject was completed by a professional neuropsychologist within 1 day of the MRI scans. General cognitive function was assessed using the Mini-Mental State Examination (MMSE) and Beijing version of the Montreal Cognitive Assessment (MoCA). Information processing speed was evaluated by the Stroop Color and Word Tests B (SCWT-B) and the Trail Making Test-A (TMT-A). The SCWT-C and TMT-B are measures of executive function. Episodic memory was evaluated by the visual reproduction–long-delayed recall portion of the Wechsler Memory Scale (WMS-VR-DR) and the Huashan version of the Auditory Verbal Learning Test-delayed recall (AVLT-DR). Language was assessed using the Category Verbal Fluency (CVF) and the Boston Naming Test (BNT). Visuospatial function was evaluated using the Clock Drawing Test (CDT) and the Visual Reproduction-copy (VR-C). It is worth noting that the results from the TMT and SCWT represent time, so they were inversely transformed to maintain consistency. The raw test scores were transformed into Z-scores and averaged to evaluate general cognitive function and function in various cognitive domains (Chen et al., 2019a). In addition, anxiety and depressive disorders were represented by the Hamilton Anxiety Scale (HAMA) and Hamilton Depression Scale (HAMD), respectively. The Timed Up and Go (TUG) test and the Tinetti Balance and Gait Analysis evaluated the fall risk and gait disorders of each subject, respectively. The Z-scores were shown in **Table 3**, and the raw scores were shown in Supplementary Table 2.

Subjects with CI were distinguished by the MMSE and MoCA scores and their educational experience. The cut-offs of CI for MMSE and MoCA were made based on the studies conducted among the Chinese population. The subject was defined as cognitive impaired when his scores were equal to or lower than the cut-off values of MMSE or MoCA (Gu et al., 2019). (The cut-offs are shown in Supplementary Table 1).

### Statistical Analysis

Demographics, clinical information, and neuropsychological data from the HC and CSVD groups were analyzed using SPSS 22.0 (Chicago, IL, USA). The comparisons between normally distributed continuous variables were performed by two sample *t*-tests, and Mann-Whitney *U*-tests were applied to compare the non-normally distributed neuropsychological and demographic data. In addition, chi-squared (χ2) tests were used in the comparisons of categorical data, such as sex and the existence of hypertension. The threshold for statistical significance was set at *p* < 0.05.

To compare the group differences in tract profiles (FA and MD), pointwise analyses were performed based on the “Randomize” program (tool in the FSL software). Age, sex, years of education, and other demographic data that varied between the groups, such as the existence of hypertension and history of LI/ transient ischemic attack (TIA), were included as covariates in the general linear model (GLM). The between-group difference at each node was tested using a permutation-based statistical analysis with 5,000 random permutations. Significance was set at *p* < 0.05 with a family-wise error (FWE) correction (Sun et al., 2015), and only differences that included three or more adjacent nodes along a tract are reported (Banfi and Koschutnig, 2019).

Then, we calculated the average FA and MD values of 100 nodes along the fiber tracts with differentiated fiber segments to explore different WM fibers between the groups from the level of fiber bundles.

Next, the partial correlations controlling for age, sex, years of education, existence of hypertension, and history of LI/TIA were applied in the CSVD group to assess the relationships between neurofunctional assessments, such as cognitive domain measurements, and all other variables of interest, including WMH volume, gray matter volume, LI count, and tract properties that differed across the groups. Bonferroni correction was conducted to adjust the false-positive rate (*p* < 0.05/27). To determine whether the integrity of the segmental fiber microstructure was related to the multidimensional clinical symptoms independent of macrostructural burden and to build predictive models of cognitive functions, a multiple linear regression analysis was performed in the CSVD patients. The cognitive domain function was taken as the dependent variable, and the significant factors in the correlation analysis were taken as the independent variables controlling for age, sex, years of education, existence of hypertension, and history of LI/TIA. All tests were two tailed, and the threshold was *p* < 0.05.

## Results

### Demographic and Neuropsychological Characteristics

The demographic and clinical data of the HC and CSVD groups are presented in Table 2. There was no significant difference in sex (*p* = 0.742) or years of education (*p* = 0.059). The individuals in the CSVD group were significantly older than those in the HC group. When vascular risk factors were compared between the two groups, the CSVD group showed a significantly increased history of hypertension (*p* = 0.001) and LI/TIA (*p* < 0.001). Thus, age, history of hypertension, and history of LI/TIA were employed as the covariates in the subsequent analyses.

**Table 2:** Demographic and clinical characteristics.

	**HC(*n* = 100)**	**CSVD(*n* = 144)**	**F/χ2/Z**	***p*-value**
Age, years	60.48 ± 7.82	65.75 ± 8.35	0.358	0.000^*^
Gender(male/female)	53/47	74/70	0.108	0.742
Education, years	11.80 ± 3.77	10.89 ± 3.57	0.076	0.059
Hypertension, *n*(%)	47(47)	98(68.10)	10.851	0.001^*^
Diabetes Mellitus, *n*(%)	18(19.40)	41(29.10)	2.810	0.094
Hyperlipidemia, *n*(%)	18(19.80)	31(22.50)	0.235	0.628
Coronary heart disease, *n*(%)	4(4.40)	9(6.60)	0.481	0.488
History of LI/TIA, *n*(%)	11(11)	47(32.60)	15.250	0.000^*^
History of smoking, *n*(%)	21(23.10)	31(23.10)	0.000	0.992
History of drinking, *n*(%)	19(21.10)	27(20.30)	0.022	0.883
Adjusted WMH volume, ×100%, *M*(IQR)	0.11(0.08~0.14)	0.36(0.23~0.57)	55.044	0.000^*^
Adjusted gray matter, ×100%	41.06 ± 1.15	39.98 ± 1.56	7.907	0.000^*^
LI count, M(IQR)	0(0~0)	1.00(0.00~2.00)	−9.237	0.000^*^
Microbleeds, M(IQR)	0(0~0)	0(0.00~2.00)	−5.2020	0.000^*^

HC, health control; CSVD, cerebral small vascular disease; n, number; TIA, transient ischaemic attack; M, mean; IQR, interquartile range; WMH, white matter hyperintensities; LI, lacunar infarction.

Values are presented as the mean ± standard deviation (SD), n (%), or M (IQR).

* *indicates a statistical difference between groups, p < 0.05*.

LI lesions were present in 59% of the enrolled CSVD patients and nearly 80.6% of the patients presented with WMH. As expected, compared with the HCs, the CSVD patients had more extensive WMH volumes (*p* < 0.001), a greater number of LIs (*p* < 0.001), and a significantly reduced volume of gray matter (*p* < 0.001). Among the CSVD group, 66 subjects were cognitively normal, accounting for 45.8%, and nearly 54.2% were those with CI. Compared with the HC group, the CSVD group also showed poor performance in the general cognitive function and all of the cognitive domains, including information processing speed, executive function, episodic memory, language, and visuospatial function. In addition, they obtained higher scores on the HAMA and HAMD, and significant differences in the two gait evaluations were shown between the two groups. The detailed neuropsychological characteristics are shown in Table 3.

**Table 3:** Z-scores of neuropsychological measurement.

	**HC(*n* = 100)**	**CSVD(*n* = 144)**	**F/Z**	***p*-value**
Z-General cognitive function	0.56(0.16~0.84)	−0.12(−0.81~0.53)	−6.342	0.000^*^
Z-MMSE	0.41(−0.13~0.95)	−0.13(−0.67~0.41)	−3.601	0.000^*^
Z-MoCA	0.74(0.18~1.02)	−0.10(−0.94~0.46)	−7.07	0.000^*^
Z-Information Processing Speed	0.17(−0.08~0.87)	−0.36(−0.74~0.17)	−6.023	0.000^*^
Z-TMT-A (inverse)	0.28(−0.32~0.87)	−0.55(−0.85~0.05)	−5.984	0.000^*^
Z- SCWT-B (inverse)	0.16(−0.30~0.86)	−0.29(−0.81~0.54)	−4.015	0.000^*^
Z-Executive Function	0.26(−0.18~0.77)	−0.38(−0.74~0.17)	−5.848	0.000^*^
Z-TMT-B (inverse)	0.28(−0.23~1.15)	−0.53(−0.89~0.09)	−6.499	0.000^*^
Z- SCWT-C (inverse)	−0.11(−0.44~0.71)	−0.38(−0.68~0.43)	−2.589	0.010^*^
Z-Episodic Memory	0.34 ± 0.72	−0.24 ± 0.83	0.572	0.000^*^
Z-AVLT-DR	0.33(−0.28~0.68)	−0.23(−0.76~0.20)	−4.987	0.000^*^
Z- VR-DR (WMS)	0.35(−0.24~0.88)	−0.24(−0.79~0.53)	−4.86	0.000^*^
Z-Language	0.23 ± 0.72	−0.16 ± 0.87	1.903	0.000^*^
Z-CVF	0.04(−0.54~0.45)	−0.09(−0.79~0.45)	−2.714	0.007^*^
Z-BNT	0.42(−0.06~0.91)	−0.23(-0.70-0.54)	−4.773	0.000^*^
Z-Visuospatial Function	0.42(0.18~0.42)	0.42(−0.37~0.42)	−2.386	0.017^*^
Z-VR-C	0.32(0.21~0.32)	0.32(−0.12~0.32)	−1.964	0.05
Z-CDT	0.51(0.19~0.51)	0.51(−0.13~0.51)	−2.383	0.017^*^
Z-HAMA	−0.55(−0.86~0.21)	−0.10(−0.63~0.97)	−3.989	0.000^*^
Z-HAMD	−0.29(−0.95~0.37)	−0.29(−0.73~0.81)	−2.079	0.038^*^
Z-Tinetti Balance Analysis	0.30(0.21~0.30)	0.30(−0.15~0.30)	−1.943	0.052
Z-Tinetti Gait Analysis	0.28(0.25~0.28)	0.28(−0.17~0.28)	−2.015	0.044^*^
Z-TUG	0.00(−1.01~0.06)	−0.15(−0.59~0.00)	−7.451	0.000^*^

HC, health control; CSVD, cerebral small vascular disease; n, number; MMSE, Mini-Mental State Examination; TMT-A and TMT-B, Trail Making Test-A and B; SCWT-B and C, Stroop Color and Word Tests B and C; AVLT-DR, Auditory Verbal Learning Test-delayed recall; VR-DR (WMS), visual reproduction–long-delayed recall portion of the Wechsler Memory Scale; CVF, Category Verbal Fluency; BNT, Boston Naming Test; VR-C, Visual Reproduction-copy; CDT, Clock Drawing Test; HAMA, Hamilton Anxiety Scale; HAMD, Hamilton Depression Scale; TUG, Timed Up and Go Test.

Valus are presented as the M ± SD or M (IQR).

* *indicates a statistical difference between groups, p < 0.05*.

### Fiber Tracking and Group Comparisons at Pointwise Levels

Using the AFQ, we successfully identified 16 fiber tracts in both of the groups, including the bilateral anterior thalamic radiation (ATR), bilateral corticospinal tract (CST), bilateral cingulum cingulate (CC), callosum forceps major, callosum forceps minor, bilateral inferior fronto-occipital fasciculus (IFOF), bilateral inferior longitudinal fasciculus (ILF), bilateral superior longitudinal fasciculus (SLF), and bilateral uncinated fasciculus (UF). Pointwise alterations in FA and MD were analyzed between the groups.

**Figure 1:**
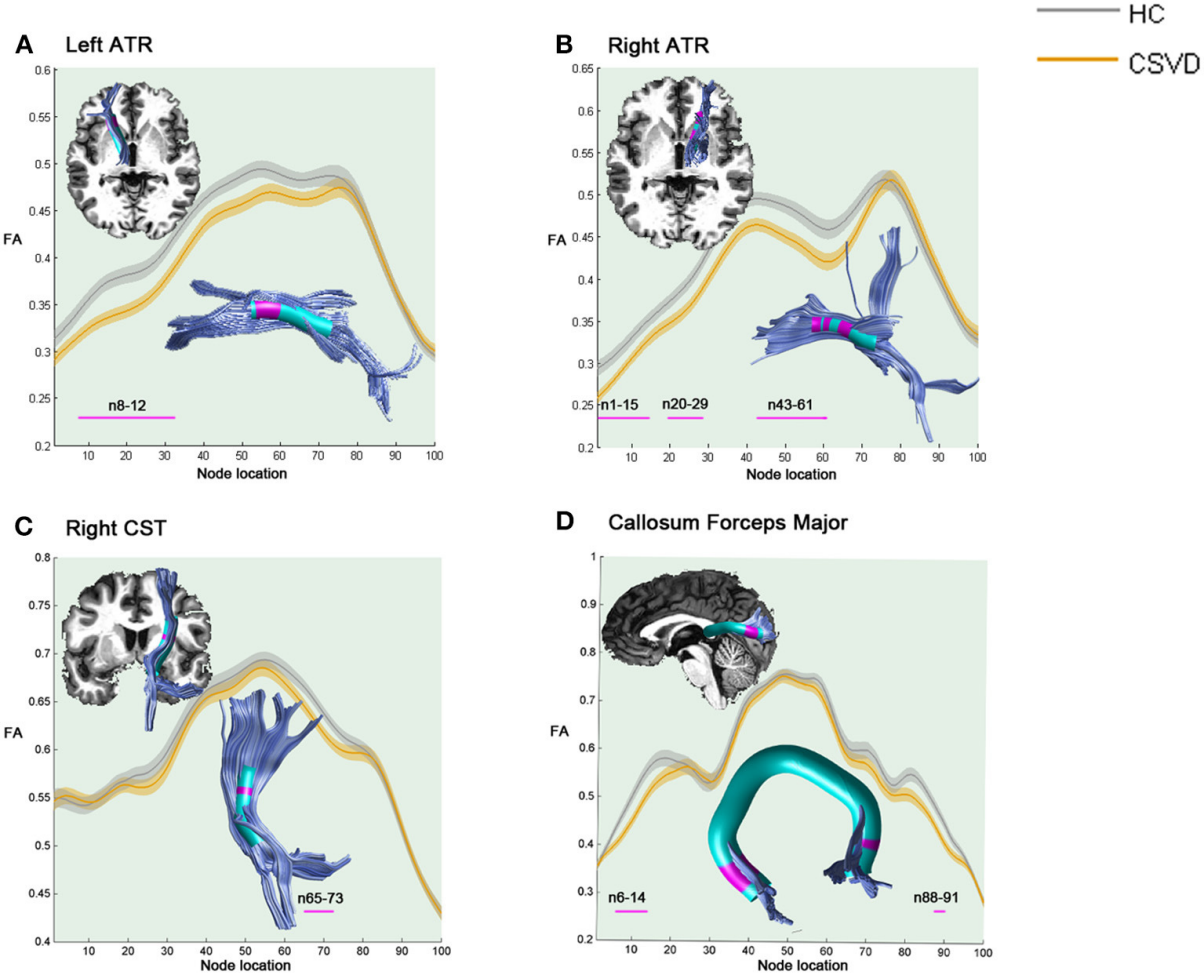
Significantly damaged locations of fiber tracts between the HC and CSVD groups in the pointwise comparison of FA profiles (FWE correction, *p* < 0.05). The blue line indicates the HC group, the orange line indicates the CSVD group, the shadows represent the confidence interval, and the pink fiber segments are the position of the fiber segment that is different between the groups. **(A)** Significantly decreased FA values in the anterior component of the left ATR (nodes 8–32) in CSVD group. **(B)** Significantly decreased FA values in the anterior and intermediate components of the right ATR (nodes 1–15; nodes 20–29; and nodes 43–61) in CSVD group. **(C)** Significantly decreased FA values in the superior portion of the right CST (nodes 65–73) in CSVD group. **(D)** Significantly decreased FA values in the occipital lobe portions of the callosum forceps major (nodes 6–14 and nodes 88–91) in CSVD group. HC, health control; CSVD, cerebral small vessel disease; FA, fractional anisotropy; ATR, anterior thalamic radiation; CST, corticospinal tract.

As shown in Figure 1, compared with HCs, the CSVD individuals demonstrated significantly decreased FA in the anterior component of the left ATR (nodes 8–32), the anterior and intermediate components of the right ATR (nodes 1–15; nodes 20–29; and nodes 43–61), the superior portion of the right CST (nodes 65–73), and the occipital lobe portions of the callosum forceps major (nodes 6–14 and nodes 88–91).

**Figure 2:**
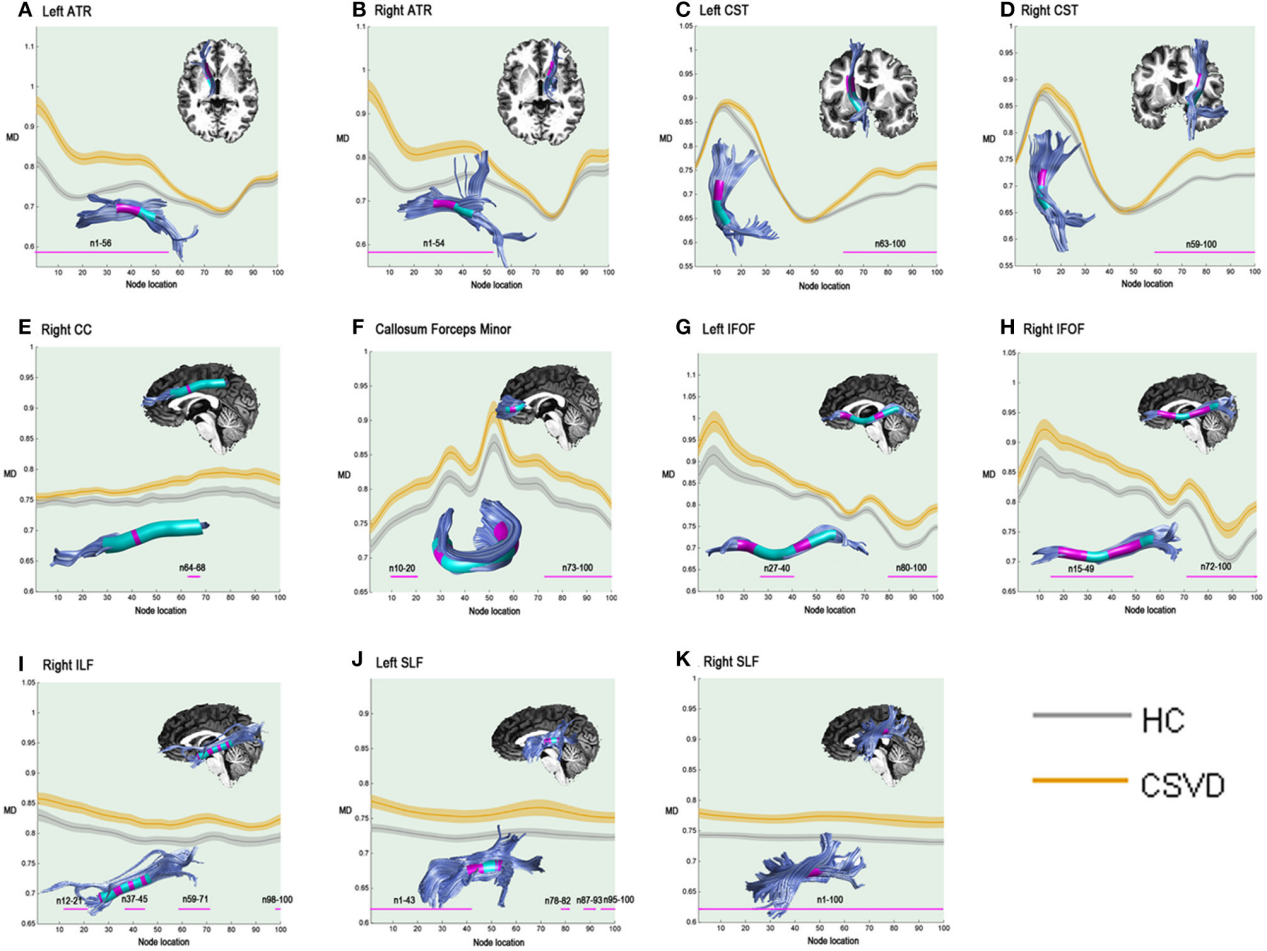
Significantly damaged locations of fiber tracts between the HC and CSVD groups in the pointwise comparison of MD profiles (FWE correction, *p* < 0.05). The blue line indicates the HC group, the orange line indicates the CSVD group, the shadows represent the confidence interval, and the pink fiber segments are the position of the fiber segment that is different between the groups. The significantly increased MD values in CSVD group (FWE correction, *p* < 0.05) were located in **(A)** the anterior half of the left ATR (nodes 1–56); **(B)** the anterior half of the right ATR (nodes 1–54); **(C)** the superior portion of the left CST (nodes 63–100); **(D)** the superior portion of the right CST (nodes 59–100); **(E)** the posteromedial component of the right CC (nodes 64–68); **(F)** the frontal lobe portions of the callosum forceps minor (nodes 10–20 and nodes 73–100); **(G)** the anterior and intermediate portions of the left IFOF (nodes 27–40 and nodes 80–100); **(H)** the anterior and intermediate components of the right IFOF (nodes 15–49 and nodes 72–100); **(I)** the anterior, intermediate and posterior portions of the right ILF (nodes 12–21; nodes 37–45; nodes 59–71; and nodes 98–100); **(J)** the anterior and posterior portions of the left SLF (nodes 1–43; nodes 78–82; nodes 87–93; and nodes 95–100); and **(K)** the entire fiber bundle of the right SLF (nodes 1–100). HC, health control; CSVD, cerebral small vessel disease; MD, mean diffusivity; ATR, anterior thalamic radiation; CST, corticospinal tract; CC, cingulum cingulate; IFOF, inferior fronto-occipital fasciculus; ILF, inferior longitudinal fasciculus; SLF, superior longitudinal fasciculus.

In the pointwise comparison of the MD properties, the significantly damaged locations in the fiber tracts (FWE correction, *p* < 0.05) are shown: (1) the anterior half of the left ATR (nodes 1–56); (2) the anterior half of the right ATR (nodes 1–54); (3) the superior portion of the left CST (nodes 63–100); (4) the superior portion of the right CST (nodes 59–100); (5) the posteromedial component of the right CC (nodes 64–68); (6) the frontal lobe portions of the callosum forceps minor (nodes 10–20 and nodes 73–100); (7) the anterior and intermediate portions of the left IFOF (nodes 27-40 and nodes 80–100); (8) the anterior and intermediate components of the right IFOF (nodes 15–49 and nodes 72–100); (9) the anterior, intermediate and posterior portions of the right ILF (nodes 12–21; nodes 37–45; nodes 59–71; and nodes 98–100); (10) the anterior and posterior portions of the left SLF (nodes 1–43; nodes 78–82; nodes 87–93; and nodes 95–100); and (11) the entire fiber bundle of the right SLF (nodes 1–100). The segments with damaged MD values are shown in Figure 2.

### Group Difference in WM Tract Level

Then, when we compared the diffusion metrics in the WM tract level, the mean FA values of the right CST were no longer different between the groups, whereas other tracts remained significantly different (details in Supplementary Figures 1, 2).

### Relationships Between Conventional MRI Markers of CSVD and Neuropsychological Measurements in CSVD Group

**Figure 3:**
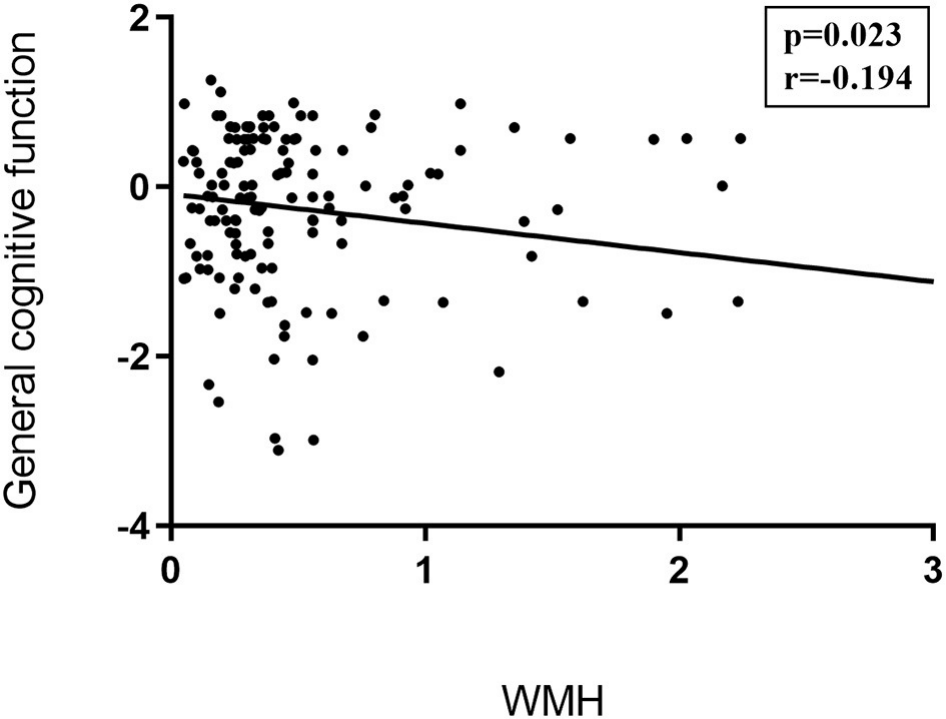
Correlations between traditional CSVD image markers and neuropsychological examinations. Partial correlation was conducted controlling for age, gender, years of education, history of hypertension, and history of LI/TIA in patients with CSVD. General cognitive function was negatively associated with WMH (*r* = −0.194, *p* = 0.023). CSVD, cerebral small vessel disease; LI, lacunar infarction; TIA, transient ischemic attack;WMH, white matter hyperintensities.

Partial correlations were performed between the cognitive domains tests/emotional assessments/gait evaluations and the conventional MRI markers like corrected WMH volumes, gray matter volumes, and LI counts. We only found that general cognitive function was negatively associated with WMH (*r* = −0.194, *p* = 0.023; details in Figure 3).

### Relationships Between Diffusion Metrics and Neuropsychological Measurements in CSVD Group

Partial correlations controlling for age, sex, years of education, history of hypertension, and history of LI/TIA were performed between the cognitive domains tests/emotional assessments/gait evaluations and mean diffusion metrics (FA or MD) in each fiber cluster where group differences emerged.

**Figure 4:**
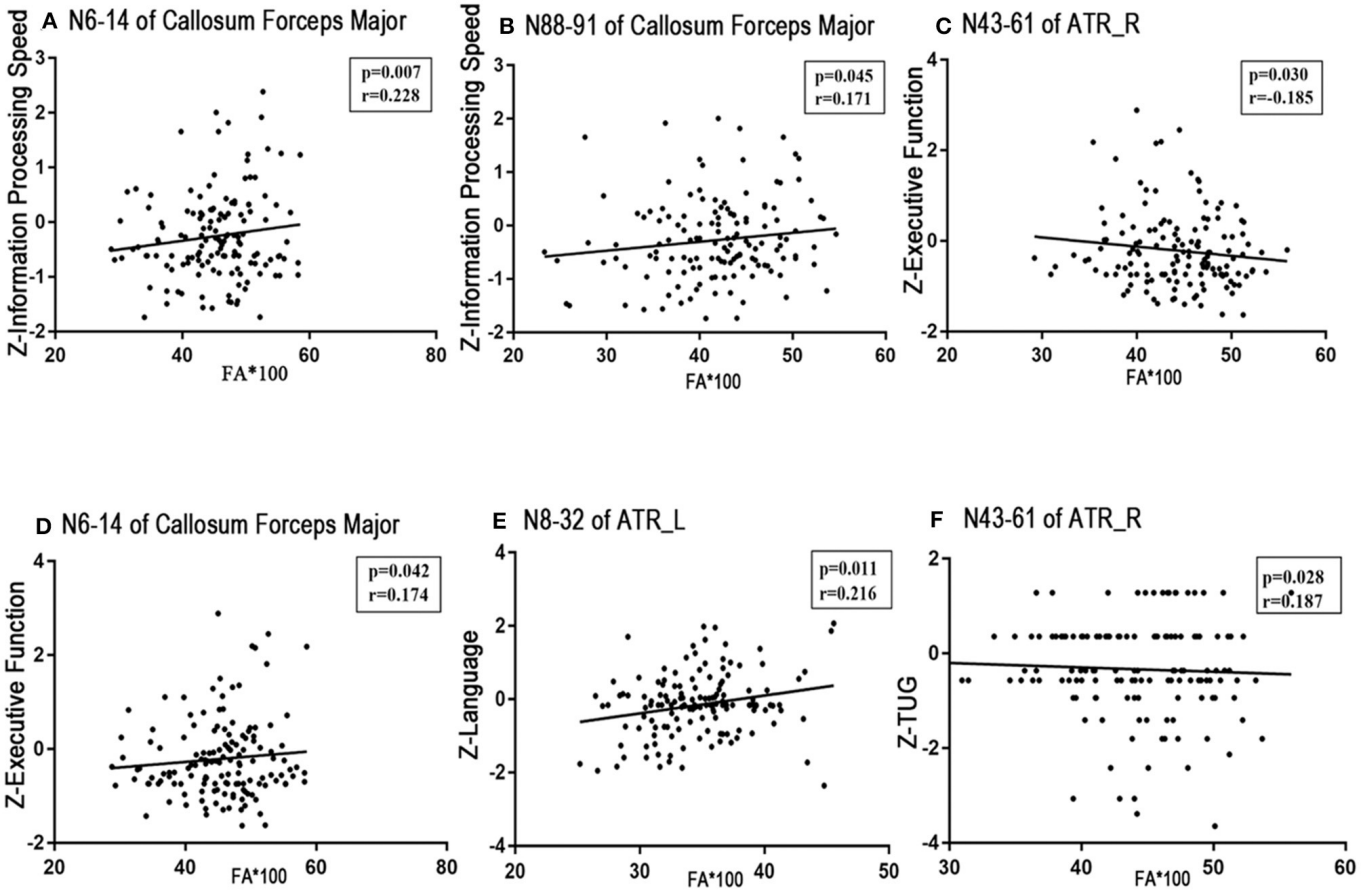
Correlations between FA profiles and neuropsychological examinations. Partial correlation was conducted controlling for age, gender, years of education, history of hypertension, and history of LI/TIA in patients with CSVD. **(A,B)** Information processing speed was positively correlated with the mean FA values of the occipital lobe portion of the callosum forceps major (nodes 6–14, *r* = 0.228, *p* = 0.007; nodes 88–91, *r* = 0.171, *p* = 0.045). **(C,D)** Executive function was negatively related to the FA value of the intermediate component of the right ATR (nodes 43–61, *r* = −0.185, *p* = 0.030), whereas was positively correlated with the FA of the occipital lobe portion of the callosum forceps major (nodes 6–14, *r* = 0.174, *p* = 0.042). **(E)** Language function was positively correlated with the FA of anterior component of the left ATR (nodes 8–32, *r* = 0.216, *p* = 0.011). **(F)** TUG showed positive correlated with the FA value of the intermediate component of the right ATR (nodes 43–61, *r* = 0.187, *p* = 0.028). CSVD, cerebral small vessel disease; LI, lacunar infarction; TIA, transient ischemic attack; WMH, white matter hyperintensities; ATR, anterior thalamic radiation.

**Figure 5:**
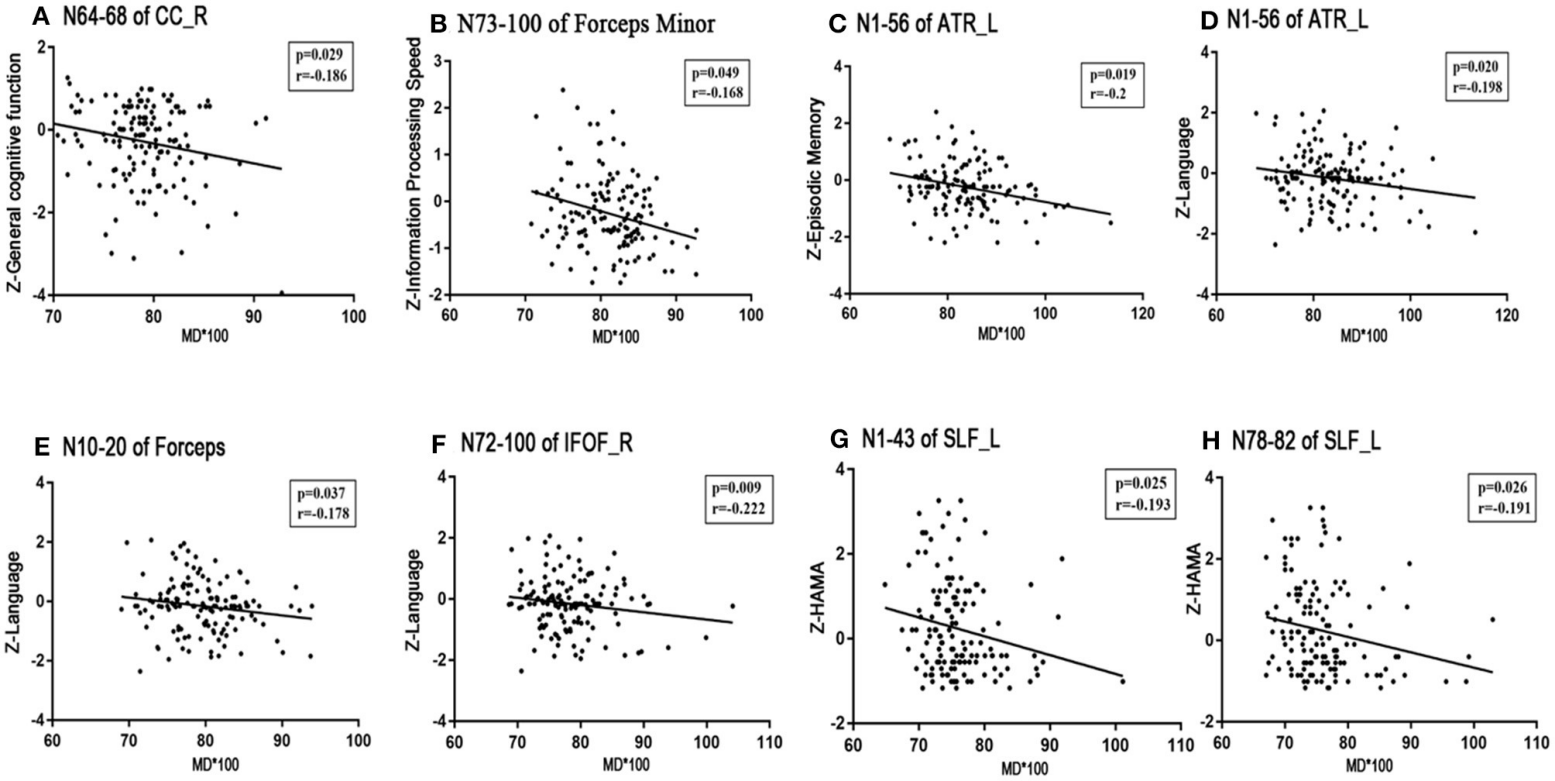
Correlations between MD profiles and neuropsychological examinations. Partial correlation was conducted controlling for age, gender, years of education, history of hypertension, and history of LI/TIA in patients with CSVD. **(A)** The mean MD value of the posteromedial component of the right CC (nodes 64–68) correlated negatively with general cognitive function (*r* = −0.186, *p* = 0.029). **(B)** The mean MD value of the frontal lobe portion of the callosum forceps minor (nodes 73–100) correlated negatively with information processing speed (*r* = −0.168, *p* = 0.037). **(C)** Episode memory was negatively associated with the MD value of the anterior half of the left ATR (nodes 1–56, *r* = −0.2, *p* = 0.019). **(D–F)** Higher MD in the anterior half of the left ATR (nodes 1–56, *r* = −0.198, *p* = 0.020), frontal lobe portion of the callosum forceps minor (nodes 10–20, *r* = −0.178, *p* = 0.037), and anterior component of the right IFOF (nodes 72–100, *r* = −0.222, *p* = 0.009) showed significant impairment in the language domain. **(G,H)** HAMA was negatively related to the MD values of the anterior and posterior portion of left SLF (nodes 1–43, *r* = −0.195, *p* = 0.025; nodes 78–82, *r* = −0.191, *p* = 0.026). CSVD, cerebral small vessel disease; LI, lacunar infarction; TIA, transient ischemic attack; WMH, white matter hyperintensities; MD, mean diffusivity; CC, cingulum cingulate; ATR, anterior thalamic radiation; IFOF, inferior fronto-occipital fasciculus; SLF, superior longitudinal fasciculus; HAMA, Hamilton Anxiety Scale.

No significant correlations existed between the visuospatial function/HAMD scores and any tract profile. We found significant negative correlations between the general cognitive function scores and the mean MD values of the posteromedial component of the right CC (nodes 64–68, *r* = −0.186, *p* = 0.029; **Figure 5A**). Information processing speeds were positively correlated with the mean FA values of the occipital lobe portions of the callosum forceps major (nodes 6–14, *r* = 0.228, *p* = 0.007; nodes 88–91, *r* = 0.171, *p* = 0.045; Figures 4A,B); and processing speeds were also negatively correlated with the mean MD values of the frontal lobe portion of the callosum forceps minor (nodes 73–100, *r* = −0.168, *p* = 0.049; Figure 5B). Significant positive correlations were observed between the executive function scores and the FA values of the occipital lobe portion of the callosum forceps major (nodes 6–14, *r* = 0.174, *p* = 0.042; Figure 4D). It was beyond our expectation that executive function scores were negatively related to the FA values of the intermediate component of the right ATR (nodes 43–61, *r* = −0.185, *p* = 0.030; Figure 4C). Higher cortical MD values in the anterior half of the left ATR (nodes 1–56) were correlated with impairments in the episodic memory (*r* = −0.2, *p* = 0.019; Figure 5C). Lower FA values in the anterior component of the left ATR (nodes 8–32, *r* = 0.216, *p* = 0.011; Figure 4E) and higher MD values in the anterior half of the left ATR (nodes 1–56, *r* = −0.198, *p* = 0.020; Figure 5D), frontal lobe portion of the callosum forceps minor (nodes 10–20, *r* = −0.178, *p* = 0.037; Figure 5E), and anterior component of the right IFOF (nodes 72–100, *r* = −0.222, *p* = 0.009; Figure 5F) showed associations with impairments in the language domain. TUG scores were positively correlated with the FA values of the intermediate component of the right ATR (nodes 43–61, *r* = 0.187, *p* = 0.028; Figure 4F). When focusing on anxiety, HAMA scores were negatively related to the MD values of the anterior and posterior portions of the left SLF (nodes 1–43, *r* = −0.195, *p* = 0.025; nodes 78–82, *r* = −0.191, *p* = 0.026; Figures 5G,H). It is worth noting that the correlations listed above were not able to survive Bonferroni correction (*p* < 0.05/27), so we chose more liberal uncorrected statistical thresholds.

Also, we summarized the correlations between the mean values of different WM fibers and the cognition assessments in Supplementary Figure 3 and detected fewer strategic WM fibers than from the pointwise level analysis. We did not find any significantly damaged WM fibers, which were related to general cognitive function, episodic memory, visuospatial function, TUG, or HAMD. We found the mean FA values of forceps major were significantly related to both executive function (*r* = 0.181, *p* = 0.034) and information processing speed (*r* = 0.259, *p* = 0.002), and the mean MD values of right CC correlated negatively with information processing speed (*r* = −0.200, *p* = 0.0.018). In addition, the mean MD values of left ATR showed an impact on the language function. Besides, only the damage to the left SLF showed an effect on the impairment of anxiety disorder (*r* = −0.192, *p* = 0.026).

### Multiple Linear Regression Analysis

The multiple regression analysis revealed that the FA value of the occipital lobe portion of the callosum forceps major, the MD value of the anterior half of the left ATR, and the FA value of the anterior component of the left ATR were independent predictors of information processing speed, episodic memory, and language domain scores, respectively. Interestingly, we also found that the FA values of the intermediate component of the right ATR were negatively related to the executive function scores but positively correlated to the TUG scores. HAMA scores were negatively related to the MD values of the posterior portion of the left SLF (details in Table 4).

**Table 4:** Multiple linear regression analysis.

	**β**	***P***
Z-Information Processing Speed (inverse)		
Education	0.456	0.000
Age	−0.284	0.000
FA value of N6-14 of Forceps Major	0.169	0.018
Z-Executive Function (inverse)		
Education	0.379	0.000
Age	−0.323	0.000
FA value of N43-61 of ATR_R	−0.207	0.006
FA value of N6-14 of Forceps Major	0.155	0.036
Z-Episodic Memory		
Education	0.214	0.008
MD value of N1-56 of ATR_L	−0.257	0.002
Z-Language		
Education	0.394	0.000
Gender	−0.204	0.006
FA value of N8-32 of ATR_L	0.193	0.010
HAMA		
Age	−0.174	0.039
Gender	0.164	0.046
MD value of N78-82 of SLF_L	−0.178	0.035
TUG		
Age	0.351	0.000
History of TIA/LI	0.241	0.002
FA value of N43-61 of ATR_R	0.161	0.041

*WMH, white matter hyperintensities; MD, mean diffusivity; FA, fractional anisotropy; ATR_L, left anterior thalamic radiation; ATR_R, right anterior thalamic radiation; SLF_L, left superior longitudinal fasciculus; HAMA, Hamilton Anxiety Scale*.

## Discussion

In this study, for the first time, we found specific changes in the microstructural integrity in patients with CSVD by AFQ: (1) WM microstructural damage presented in an extensive interhemispheric symmetrical pattern; (2) the damage did not occur along the entire trajectory of a WM fascicle but along some vulnerable fibrous segments. The pointwise level analysis is more sensitive than the analysis in the WM fiber level where more damaged fiber segments can be found, and more microstructural damages affecting clinical cognition can be detected; and (3) impairments in the different WM portions might mediate different cognitive domain dysfunction, but the anterior middle portion of the ATR causes impairments in multiple recognition domains. These results suggested that the segmental WM fascicle microstructural damage detected by the AFQ may be a marker for the early prediction of CSVD-related cognitive decline, revealing the pathological mechanism of CSVD-related CI from an anatomical perspective.

We first studied the WM microstructural integrity between the CSVD patients and the HCs using the AFQ, which included both the WMH and the normal appearing WM on MRI. The AFQ is a more sensitive tractography method that allows the identification of a complete map of the distribution of cerebral damage in those with CSVD at the individual level. As diffusion properties always vary along the tract trajectory, the AFQ provides a framework for quantifying diffusion metrics at multiple nodes along the trajectory of each fiber tract, generating the “tract profile” (Yeatman et al., 2012). In this study, we observed that the CSVD patients exhibited a significantly damaged tract profile along the multiple bundles, with FA decreased in 25% (4/16) and MD increased in 68.75% (11/16) of the fiber tracts examined. These tracts belong to three fiber systems, namely, the association WM fibers (CC, IFOF, ILF, and SLF), commissural WM ROIs (callosum forceps major and callosum forceps minor), and projection WM fibers (CST and ATR). When compared from the WM fiber level, fewer damaged fibers were found, and that is one of the advantages of AFQ that exploration at the node level can reduce the coverage of some small damage clusters (Chen et al., 2020). As Figures 1, 2 show, most of the microstructural damage showed interhemispheric symmetry and heterogeneity, and this pattern was partly in accordance with macroscopic WMH on T_2_-weighted images in the CSVD patients (Pantoni, 2010). Mascalchi et al. (2017) focused on CADASIL, an inherited CSVD, and declared an extensive symmetric pattern of fiber damage by DTI analysis. These similar results may be due to extensive neuropathological changes in the leptomeningeal and small penetrating cerebral arteries, such as the loss of smooth muscle cells in the tunica media, deposits of fibrohyaline materials, and thickening of the vessel wall with incomplete lumen occlusion (Pantoni, 2010; Mascalchi et al., 2017). In addition, we found that the diffusion properties varied along the WM fascicle trajectory in the CSVD patients and that there existed some vulnerable nodes or segments along a fiber, consistent with the findings on Alzheimer's disease (Zhang et al., 2019).

Exhilaratingly, this study found that specific WM fascicle segments were related to particular brain functions, such as cognition, emotion, and gait. And compared with strategic WM fibers, more strategic segments were detected, which may have an impact on clinical manifestations. In particular, these segmental correspondences to particular cognitive domains were identified through the multiple linear regression analysis (Table 4). In other words, the specific WM fascicle segment might mediate the corresponding brain dysfunction if the WM microstructure was damaged in CSVD patients.

First, this study found that with the damage of the microstructure of nodes 64–68 in the right CC, the general cognitive function of CSVD patients tended to decline. The cingulum bundle has been implicated in episodic memory, executive control, emotion, and pain. Numerous studies have revealed that cingulum abnormalities could lead to Alzheimer's disease, schizophrenia, depression, posttraumatic stress disorder, and mild cognitive impairment (MCI; Bubb et al., 2018). Our results are supported by some brain perfusion and metabolism studies by PET, that declared reduced metabolism in a network of limbic structures such as the posterior cingulate in MCI patients (Nestor et al., 2003). A resting-state functional MRI study also found that the posterior cingulate cortex is the main connecting hub of the main cortical networks (Buckner et al., 2009).

Second, this study showed that there was a positive relationship between the FA values of nodes 6–14 of the forceps major and information processing speed or executive function. FA was most commonly reported to be associated with processing speed in the genu and body of the corpus callosum in elderly adults (Kerchner et al., 2012). Our findings highlight the occipital lobe portion nodes 6–14 of the callosum forceps major as an anatomical structure potentially impacting processing speed. This is partly supported by Duering et al., who identified this area as strategic fibers affecting processing speed in patients with CADASIL (Duering et al., 2011). We further proposed that segmental damage of the key fiber bundles might be sufficient to reduce information processing speeds due to the disconnection of interhemispheric information transmission.

Notably, the present study first showed that different WM fascicle segments of the ATR might be related to different cognitive domains. The MD values of nodes 1–56 (anterior half) of the left ATR were an independent predictor of episodic memory scores. The FA values of nodes 8–32 (anterior portion) of the left ATR were independently related to language function, and the FA values of nodes 43–61 (medium portion) of the right ATR were independently related to both executive and gait functions.

The ATR begins in the anterior and mediodorsal nuclei of the thalamus and projects to the anterior cingulate cortex and dorsolateral frontal regions (Mamiya et al., 2018), being the core structure of frontothalamic circuitry. Studies have shown that lesions to the anterior nuclei or their afferent WM tracts result in deficits in the encoding of new stimuli and thus affect the declarative memory process (Van Der Werf et al., 2003). The anterior nuclei are involved in the selection of memory materials, and the mediodorsal nucleus is responsible for coordinating and selecting strategies to retrieve declarative memory (Van Der Werf et al., 2003). A PET analysis in those with vascular dementia exhibited a hypometabolic pattern in the frontothalamic circuitry, which was related to memory disruptions (Longarzo et al., 2019). In addition, hypometabolism in patients with amnestic-MCI occurs mainly in the left hemisphere (Seo et al., 2009). Asymmetry in cortical activation has also been suggested, for example, language-based working memory tasks preferentially activated the left hemisphere (O'Sullivan et al., 2005), which is consistent with our results that only the left ATR was related to memory. Our results may also serve as a support for the existence of multiple memory systems. The concept of an “extended hippocampal system” composed of the hippocampus, mammillary bodies, fornix, and anterior thalamic nuclei was proposed by Aggleton and Saunders (1997). The disruption of these systems leads to deficits in the recall of episodic events, and connections between the thalamus and cortical structures were proposed to be involved in memory processing (Alexinsky, 2001). Disconnections between the prefrontal cortex and the mediodorsal or anterior thalamic nuclei might represent the pathophysiological substrates for aberrant WM microstructure in CSVD-related cognitive decline. Notably, our results emphasized the importance of the integrity of prefrontal-subcortical loop in memory management in patients with CSVD.

In addition, recent research has established that widely distributed, isolated, and shared cerebral regions are involved in both phonological and semantic fluency (Li et al., 2017). These regions are located in the left thalamus, frontal lobe, and parietal lobe (Li et al., 2017). When one produces words in response to particular cues, the left thalamus is activated. The left prefrontal lobe was reported to be involved in category fluency processing (Birn et al., 2010). Therefore, it is not surprising to find that the left ATR, which connects these key brain regions, contributes to language dysfunction (Han et al., 2013). It is also worth noting that the verbal memory and attention/executive control function are the basis of semantic and phonological fluencies (Li et al., 2017). Given the role of the ATR in memory and executive functions, the disconnection of the anterior component of the left ATR could lead to disruptions in verbal fluency (Li et al., 2017).

The dorsolateral prefrontal cortex (DLPFC) is a hub in the executive network, and functional and structural abnormalities in this network contribute to both executive impairments and decreased processing speed (Cummings, 1993; Poole et al., 2018). Ping et al. reported that the integrity of WM fibers connecting the anterior cingulate cortex and the frontal regions was related to test scores in attention; this correlation only existed in the right hemisphere (Mamiya et al., 2018). The involvement of frontothalamic projection fiber tracts in the executive function has been well-established (Tekin and Cummings, 2002) and is in line with our result of the impact of ATR on the executive function and gait evaluation. However, the FA values in the right ATR were negatively related to the executive function and gait. In other words, poor executive function or gait was related to more intact fibers in the right ATR. Some possible explanations were available to expound this seemingly conflicting trend. First, we speculated that the reason for this phenomenon was the plasticity and compensatory effects of WM fibers. Well-organized brain networks have the capacity to adapt to local deficits, and reconstruction of cellular elements within the affected networks after stroke has been reported. For example, the remodeling of functional connections in the photothrombotic infarcts in the murine cortex is often accompanied by dendritic spine turnover and the formation of new structural connections (Brown et al., 2009). The plasticity of fiber microstructure can be compensated temporarily, and we hypothesized that decompensation may occur with the further deterioration of patients' cognitive function, and this definitely needs to be confirmed by our follow-up studies in a larger sample. Next, structural damage may be accompanied by enhanced functional connectivity, which can enhance one's cognition (Chen et al., 2019b). This of course needs further research including functional magnetic resonance. Besides, cognitive reconfiguration could be another explanation. For example, the reconfiguration of the memory process by shifting from the damaged fornix to the left parahippocampal cingulum (PHC) is another confirmation of the compensatory effects of microstructural integrity (Ray and Metzler-Baddeley, 2015). In addition, since the results cannot survive rigorous multiple comparisons, other explanations cannot be ruled out, such as an increase in the FA values caused by the degeneration of crossing-fibers in these regions. In short, a larger sample is needed to conduct a further longitudinal study to validate the abovementioned speculations.

Few fiber tracts other than the posterior portion of the left SLF were associated with emotional disorders. To our surprise, with the emotional deterioration associated with anxiety, the microstructural integrity of the left SLF increased. A study by Lai and Wu involving patients with major depressive disorders reported lower FA in the left SLF of those who were severely depressed (Lai and Wu, 2014), whereas Frodl et al. (2012) found that patients at risk for major depression showed high FA in the left SLF, in agreement with our findings. We hypothesized that the reason for this contrasting result was that the mood disorders of the CSVD subjects in our study were mild or just at risk, and the subjects were resilient. Lower MD has been associated with resilience against anxiety. Resilience to stress was related to the capacity to release corticotrophin-releasing hormone and corticosterone mediated by the expression of brain-derived neurotrophic factor (BDNF) in a negative feedback system (Charney, 2004; Taliaz et al., 2011; Frodl et al., 2012). BDNF is associated with the development of structural neural circuits that control stress adaptations (Taliaz et al., 2011). This may explain the adaptation and plasticity of the left SLF in patients at high risk for mood disorders.

We also analyzed demographic variables in the multiple linear regression model and found that the executive function, information processing speed, HAMA, and TUG scores yielded significant correlations with age, while the episodic memory, language, information processing speed, and executive function scores yielded significant correlations with education. Additionally, sex is also an independent influencing factor for language and anxiety disorders.

Age-related mechanisms of neurodegenerative diseases are impairments in the connectivity of neural networks, which include both primary cortical neuronal degeneration and slowly progressing myelin-degeneration processes such as microvascular dysfunction and β-amyloid deposits (Fellgiebel et al., 2005). As our results suggested, such functionally relevant WM impairments can be sensitively detected and quantified by the AFQ. Education is efficacious on global cognition, select cognitive domains, and psychosocial functioning in people with CI (Kessels et al., 2017; Arce Renteria and Vonk, 2019). The positive effect of education on cognition shows a kind of cognitive reserve function. The cognitive reserve obtained from education cannot protect the brain from pathological changes caused by advanced age but can regulate the relationship between pathology and pathological expression. In other words, cognitive reserve is a kind of accumulation of experience and ability, which may have a positive impact on brain growth, compliance, and compensatory flexibility in the face of injury (Brickman et al., 2011). Studies have confirmed that women have a higher lifetime prevalence rate of emotional and anxiety disorders, which may be related to physiological factors such as sex hormones and, of course, social factors (Riecher-Rossler, 2017). The sex differences in patients with CSVD needs further stratified analyses.

The presence of many macrostructural lesions, measured as WMH volume and LI counts, was not significantly correlated with cognitive domains such as executive function. It should be noted that our study included the spectrum of CSVD patients, and the global dysfunction of the sample was mild. Thus, DTI measures were more sensitive in detecting WM changes and displayed close associations with cognitive function. Microstructural damage detected by DTI precedes the development of WMH on T2-W FLAIR MRI. DTI parameters are more sensitive and serve as early markers of microstructural neurodegenerative changes in CSVD patients (Ye and Bai, 2018). It should also be noted that we included the spectrum of CSVD patients, rather than limiting the study to patients with specific diagnoses such as vascular cognitive dysfunction, with the purpose of generalizing the results to all patients with at least minimal CSVD load, regardless of the final diagnosis.

However, several limitations in this study need to be noted. First, we failed to track some fibers, especially the bilateral CH and bilateral AF, because the threshold setting in the fiber tracking makes it difficult to identify tracts adjacent to the gray matter. The failure to analyze these fibers may have resulted in the loss of important findings. Second, LI is another important clinical outcome of the CSVD, and WM damage was found to be closely related to the incidence of LI (Xu et al., 2018). We did not focus on the effects of strategically located WM fibers on the incidence and recurrence of lacunar infarction. We will follow up with these patients for future studies. Third, this was a cross-sectional study, and longitudinal studies are needed to confirm these findings, especially the changes in the WM tract profiles with the development of CSVD. Last but not the least, in this exploratory work, our microstructural damages were explored from a whole-brain perspective, which involved both macrostructural lesions and normal-appearing white matter. So we cannot confirm if the relationships between cognition and DTI metrics occur in regions outside of lesions. We are now trying to identify and delineate the lesions manually to construct a normal-appearing white matter mask, and more convincing relationships between microstructural damage and cognition will be achieved in future work.

## Conclusion

The current study demonstrated that the damage to WM fiber bundles caused by the CSVD showed extensive interhemispheric symmetry and was limited to particular segments. The intermediate component of the right ATR and the anterior half of the left ATR were strategically located fibers affecting CSVD-related CI. In addition, a conflicting trend between the integrity of right ATR and the executive function or gait needs further research, and we speculated that multiple forms of compensation may exist in the brain. These findings contribute to the understanding of the neural mechanisms underlying CI in those with CSVD and shed light on the development of novel markers of CSVD-related CI.

## Data Availability Statement

The datasets generated for this study are available on request to the corresponding author.

## Ethics Statement

The studies involving human participants were reviewed and approved by the Ethics Committee of Nanjing Drum Tower Hospital. The patients/participants provided their written informed consent to participate in this study.

## Author Contributions

LH, XC, XZ, and YX designed the study, conducted the statistical analysis, and drafted the manuscript. WS, HC, and QY helped to conduct the statistical analysis and conduct the data preprocessing. DY, ML, CL, JM, PS, HX, and BZ helped to collect the data and conduct the data preprocessing. XZ and YX revised the study and manuscript and provided the critical and intellectual content for the study. All authors contributed to the article and approved the submitted version.

## Conflict of Interest

The authors declare that the research was conducted in the absence of any commercial or financial relationships that could be construed as a potential conflict of interest.

## Supplementary Material

Supplementary Figure 1 Significantly different WM fibers between the HC and CSVD groups in comparison of mean FA profiles. **(A)** The mean FA values were significantly decreased in left ATR in CSVD group. **(B)** The mean FA values were significantly decreased in right ATR in CSVD group. **(C)** The mean FA values were significantly decreased in Callosum Forcepts Major in CSVD group. HC, health control; CSVD, cerebral small vessel disease; FA, fractional anisotropy; ATR, anterior thalamic radiation. ^*^indicates a statistical difference between groups.

Supplementary Figure 2 Significantly different WM fibers between the HC and CSVD groups in comparison of mean MD profiles. The significant increased mean MD values were located in **(A)** left ATR, **(B)** right ATR, **(C)** left CST, **(D)** right CST, **(E)** right CC, **(F)** Callosum Forcepts Minor, **(G)** left IFOF, **(H)** right IFOF, **(I)** right ILF, **(J)** left SLF, **(K)** right SLF. HC, health control; CSVD, cerebral small vessel disease; MD, mean diffusivity; ATR, anterior thalamic radiation; CST, corticospinal tract; CC, cingulum cingulate; IFOF, inferior fronto-occipital fasciculus; ILF, inferior longitudinal fasciculus; SLF, superior longitudinal fasciculus. ^*^indicates a statistical difference between groups.

Supplementary Figure 3 The correlations between the mean FA or MD values and the cognition assessment in the CSVD group. **(A)** The mean FA values of callosum forceps major were significantly related to executive function (*r* = 0.181, *p* = 0.034); **(B)** The mean FA values of callosum forceps major were significantly related to information processing speed (*r* = 0.259, *p* = 0.002); **(C)** The mean MD values of right CC correlated negatively with information processing speed (*r* = −0.200, *p* = 0.0.018). **(D)** The mean MD values of the left ATR showed an impact on the language function (*r* = 0.210, *P* = 0.014); **(E)** The mean MD values of the left SLF showed a significant negative correlation with HAMA (*r* = −0.192, *p* = 0.026).
