# Supplementary figures and images for: Early Segmental White Matter Fascicle Microstructural Damage Predicts the Corresponding Cognitive Domain Impairment in Cerebral Small Vessel Disease Patients by Automated Fiber Quantification
Source: Front Aging Neurosci. 2021 Jan 11;12:598242. doi: 10.3389/fnagi.2020.598242 (PMC7829360; doi:10.3389/fnagi.2020.598242)

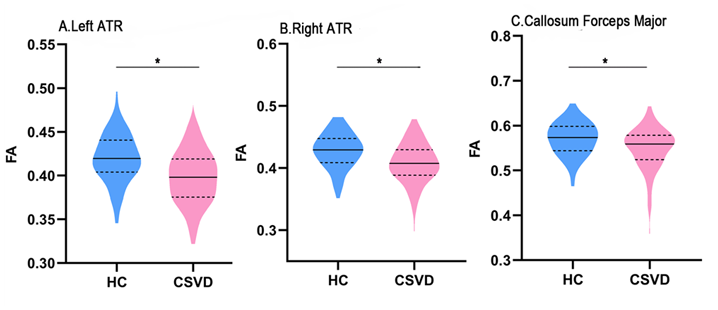

Supplement: Supplementary Figure 1 — Significantly different WM fibers between the HC and CSVD groups in comparison of mean FA profiles. (A) The mean FA values were significantly decreased in left ATR in CSVD group. (B) The mean FA values were significantly decreased in right ATR in CSVD group. (C) The mean FA values were significantly decreased in Callosum Forcepts Major in CSVD group. HC, health control; CSVD, cerebral small vessel disease; FA, fractional anisotropy; ATR, anterior thalamic radiation. *indicates a statistical difference between groups. [file Image_1.TIF]

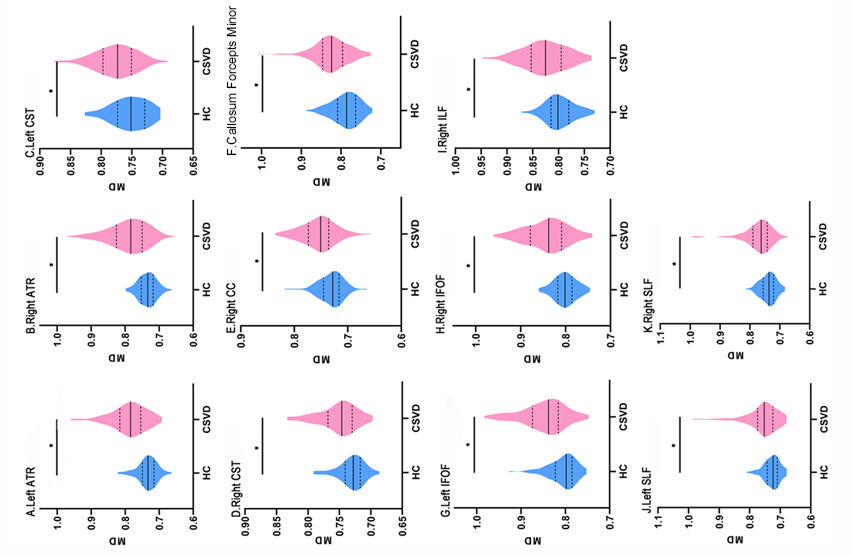

Supplement: Supplementary Figure 2 — Significantly different WM fibers between the HC and CSVD groups in comparison of mean MD profiles. The significant increased mean MD values were located in (A) left ATR, (B) right ATR, (C) left CST, (D) right CST, (E) right CC, (F) Callosum Forcepts Minor, (G) left IFOF, (H) right IFOF, (I) right ILF, (J) left SLF, (K) right SLF. HC, health control; CSVD, cerebral small vessel disease; MD, mean diffusivity; ATR, anterior thalamic radiation; CST, corticospinal tract; CC, cingulum cingulate; IFOF, inferior fronto-occipital fasciculus; ILF, inferior longitudinal fasciculus; SLF, superior longitudinal fasciculus. *indicates a statistical difference between groups. [file Image_2.tif]

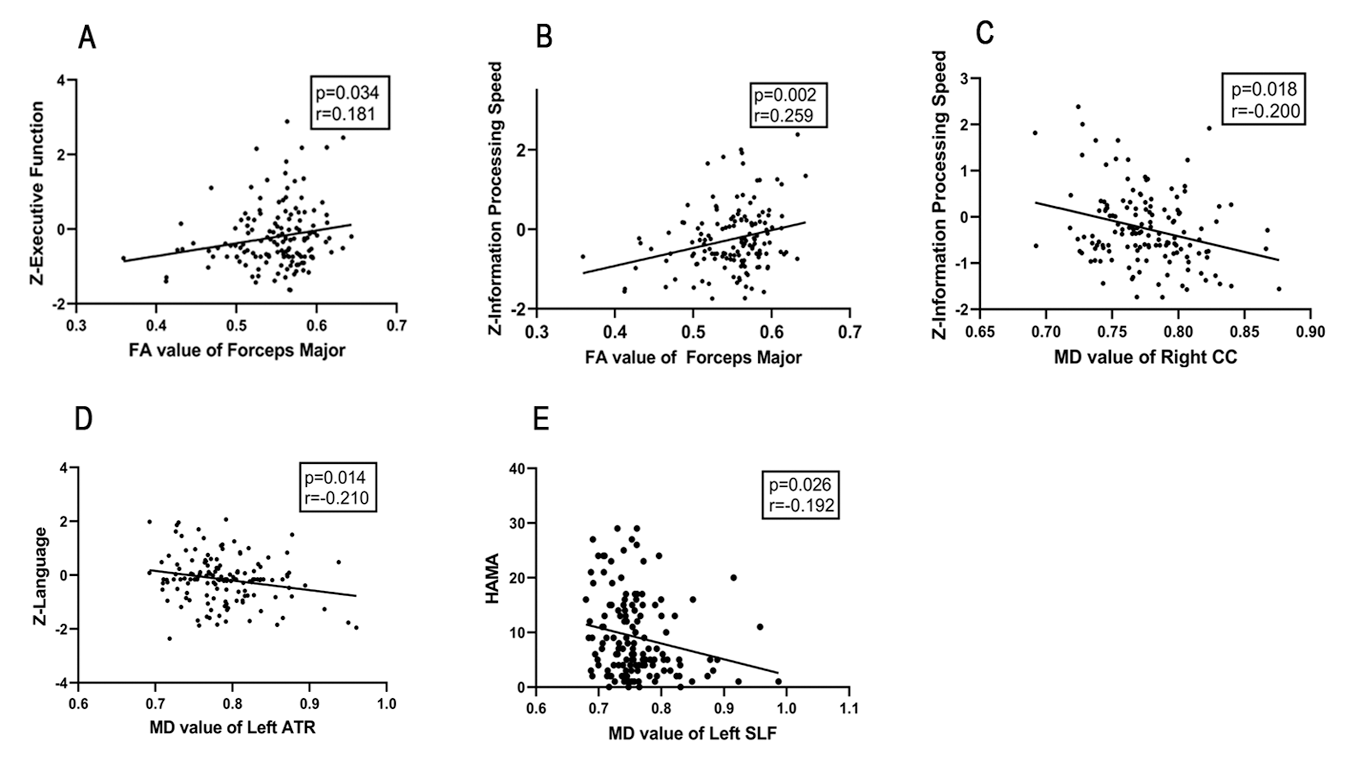

Supplement: Supplementary Figure 3 — The correlations between the mean FA or MD values and the cognition assessment in the CSVD group. (A) The mean FA values of callosum forceps major were significantly related to executive function (r = 0.181, p = 0.034); (B) The mean FA values of callosum forceps major were significantly related to information processing speed (r = 0.259, p = 0.002); (C) The mean MD values of right CC correlated negatively with information processing speed (r = −0.200, p = 0.0.018). (D) The mean MD values of the left ATR showed an impact on the language function (r = 0.210, P = 0.014); (E) The mean MD values of the left SLF showed a significant negative correlation with HAMA (r = −0.192, p = 0.026). [file Image_3.TIF]
