# Supplementary material for: Early Segmental White Matter Fascicle Microstructural Damage Predicts the Corresponding Cognitive Domain Impairment in Cerebral Small Vessel Disease Patients by Automated Fiber Quantification
Source: Front Aging Neurosci. 2021 Jan 11;12:598242. doi: 10.3389/fnagi.2020.598242 (PMC7829360; doi:10.3389/fnagi.2020.598242)
Supplement: Supplementary file 4 [file Table_1.docx]

**Supplementary Table 1 cut-offs of CI**

| Cognitive test | Education (years) | Borderlines of CI (scores) |
| --- | --- | --- |
| MMSE | 0 | ≤19 |
|  | 1-6 | ≤22 |
|  | >6 | ≤26 |
| MoCA | 0 | ≤13 |
|  | 1-6 | ≤19 |
|  | 7-12 | ≤24 |
|  | ＞12 | ≤25 |
