# Supplementary material for: Early Segmental White Matter Fascicle Microstructural Damage Predicts the Corresponding Cognitive Domain Impairment in Cerebral Small Vessel Disease Patients by Automated Fiber Quantification
Source: Front Aging Neurosci. 2021 Jan 11;12:598242. doi: 10.3389/fnagi.2020.598242 (PMC7829360; doi:10.3389/fnagi.2020.598242)
Supplement: Supplementary file 5 [file Table_2.docx]

| **Supplementary Table 2 raw scores of neuropsychological measurement** | | | | | | |
| --- | --- | --- | --- | --- | --- | --- |
|  | HC（n=100） | CSVD（n=144） | F/Z | | p value | |
| MMSE | 29.00(28.00~30.00) | 28.00(27.00~29.00) | -3.601 | | 0.000* | |
| MoCA | 27.00(25.00~28.00) | 24.00(21.00-26.00) | -7.070 | | 0.000* | |
| TMT-A (s) | 43.00(35.00~56.00) | 63.50(47.25~76.75) | -5.984 | | 0.000* | |
| SCWT-B (s) | 20.00(16.63~23.00) | 23.00(18.00~27.75) | -4.015 | | 0.000* | |
| TMT-B (s) | 82.50(58.25~109.75) | 136.00(91.00~189.97) | -6.499 | | 0.000* | |
| SCWT-C (s) | 32.00(24.00~37.00) | 36.00(26.25~41.75) | -2.589 | | 0.010* | |
| AVLT-DR | 5.27(4.00~6.00) | 4.11(3.00~5.00) | -4.987 | | 0.000* | |
| VR-DR (WMS) | 9.11(7.00~11.00) | 6.97(5.00~9.75) | -4.860 | | 0.000* | |
| CVF | 17.35(15.00~19.00) | 16.83(14.00~19.00) | -2.714 | | 0.007* | |
| BNT | 53.00(50.00~56.00) | 48.94(46.00-53.75) | -4.773 | | 0.000* | |
| VR-C | 14.00(13.91~14.00) | 14.00(13.63~14.00) | -1.964 | | 0.050 | |
| CDT | 4.00(3.81~4.00) | 4.00(3.62~4.00) | -2.383 | | 0.017* | |
| HAMA | 4.00(2.00~9.00) | 7.00(3.50~14.00) | -3.989 | | 0.000* | |
| HAMD | 4.00(1.00~7.00) | 4.00(2.00~9.00) | -2.079 | | 0.038* | |
| Tinetti Balance Analysis | 16.00(15.89~16.00) | 16.00(15.44~16.00) | -1.943 | | 0.052 | |
| Tinetti Gait Analysis | 12.00(11.97~12.00) | 12.00(11.63~12.00) | -2.015 | | 0.044* | |
| TUG(s) | 7.95(7.00~8.00) | 9.00(8.00~9.33) | -7.451 | | 0.000* | |
| Abbreviations: HC, health control; CSVD,cerebral small vascular disease;n, number;MMSE,Mini-Mental State Examination;TMT-A and TMT-B,Trail Making Test-A and B; SCWT-B and C, Stroop Color and Word Tests B and C;AVLT-DR,Auditory Verbal Learning Test-delayed recall;VR-DR (WMS),visual reproduction–long-delayed recall portion of the Wechsler Memory Scale ;CVF,Category Verbal Fluency;BNT,Boston Naming Test; VR-C,Visual Reproduction-copy;CDT,Clock Drawing Test;HAMA,Hamilton Anxiety Scale; HAMD,Hamilton Depression Scale;TUG,Timed Up and Go Test.  The raw scores of TMT, SCWT and TUG mean the time measured in seconds.  Valus are presented as the M ± SD or M (IQR).  * indicates a statistical difference between groups, p < 0.05 | | | | | | |
|  |  |  |  |  |  |  |
|  |  |  |  |  |  |  |
|  |  |  |  |  |  |  |
|  |  |  |  |  |  |  |
|  | | | |  | |  |
